# Antimalarial market composition and performance in Nigeria and the Democratic Republic of Congo: results from 2013 ACTwatch outlet surveys

**DOI:** 10.1186/1475-2875-13-S1-P57

**Published:** 2014-09-22

**Authors:** Megan Littrell, Raymond Sudoi, Julie Archer, Julius Ngigi, Chinazo Ujuju, Godéfroid Mpanya, Vamsi Vasireddy, Stephen Poyer, Hana Bilak

**Affiliations:** 1 PSI/ACTwatch, Washington DC, USA; 2 PSI/ACTwatch, Nairobi, Kenya; 3 Independent Consultant, Geneva, Switzerland; 4 Society for Family Health, Abuja, Nigeria; 5 Association de Santé Familiale, Kinshasa, The Democratic Congo; 6 Independent Consultant, Chicago, IL, USA; 7 PSI, Malaria and Child Survival Department, Nairobi, Kenya

## Background

Nigeria and the Democratic Republic of Congo (DRC) together account for one-third of the estimated global total of malaria cases and 40% of malaria deaths. Improving health system performance in these countries is critical for reducing the global malaria burden. In both countries, fever treatment-seeking in the private sector is very common. Antimalarial medicine outlet surveys conducted in Nigeria and the DRC in 2009 documented antimalarial markets dominated by informal drug stores and high non-artemisinin monotherapy (non-AMT) availability and market share, as well as moderate availability of oral AMT. 2011 findings from Nigeria showed improved artemisinin combination therapy (ACT) availability and market share, but continued availability and distribution of oral AMT. 2013 data were collected to inform and monitor ongoing strategy and funding decisions to improve malaria case management.

## Materials and methods

Antimalarial medicine outlet surveys were conducted as part of the ACTwatch project (http://www.actwatch.info) in Nigeria and the DRC in 2013. A census of all outlets with the potential to sell/distribute antimalarials was conducted within a representative sample of clusters (national in Nigeria, Kinshasa & Katanga provinces in the DRC). Drug information, sale/distribution in the previous week, and retail price were collected for every antimalarial in stock. Product and distribution information was used to calculate relative market share using the adult equivalent treatment dose as the unit of analysis.

## Results

The overwhelming majority of antimalarial-stocking outlets in the DRC and Nigeria were drug stores. In both countries, non-AMT (quinine in the DRC, chloroquine in Nigeria) and ACT were commonly in stock among antimalarial-stocking drug stores. While ACT availability was high, many drug stores in both countries were stocking non-quality-assured (QA) ACT. Oral AMT was commonly available among drug stores in Nigeria, but very rarely available in the DRC. The vast majority of antimalarials sold/distributed across study sites were distributed through drug stores. ACT (including non-QA ACT) accounted for less than half of the antimalarial market share in both countries. Non-AMT (SP, quinine, chloroquine) accounted for more than half of the market share across study sites.

## Conclusions

Informal drug stores continue to dominate the antimalarial markets in the DRC and Nigeria in terms of absolute number of service delivery points, and sale/distribution of treatments. Consumers seeking care at antimalarial-stocking drug stores will commonly find both non-AMT and ACT available. However, non-QA ACT is frequently available and distributed. Oral AMT availability continues to be of concern in Nigeria.

